# Primary and Secondary Hypogonadism in Male Persons with Diabetes Mellitus

**DOI:** 10.1155/2021/8799537

**Published:** 2021-06-04

**Authors:** João Martin Martins, Mafalda de Pina Jorge, Catarina Martins Maia, João Roque, Carlos Lemos, Daniel Nunes, Dinis Reis, Catarina Mota

**Affiliations:** ^1^Endocrine Department, Santa Maria Hospital, Lisbon, Portugal; ^2^Endocrine University Clinic, Lisbon Medical School, Lisbon, Portugal; ^3^Internal Medicine Department, Jacobi Medical Center and Albert Einstein College of Medicine, New York, NY, USA; ^4^Clinical Pathology Department, Santa Maria Hospital, Lisbon, Portugal; ^5^Internal Medicine Department, Santa Maria Hospital, Lisbon, Portugal; ^6^Internal Medicine University Clinic, Lisbon Medical School, Lisbon, Portugal

## Abstract

**Aims:**

To characterize hypogonadism in male persons with diabetes mellitus. *Patients and Methods*. 184 consecutive male persons with diabetes were studied. Besides the usual care, total testosterone (TT), estradiol (E2), FSH, and LH were measured in the last appointment and in 40 patients, also in the next two appointments. Statistical analysis compared groups and explored factors for TT and LH levels.

**Results:**

TT levels were stable and highly correlated (*r* > 0.750, *p* < 0.001) over a 6–12-month period. 20% of the patients presented secondary hypogonadism (SH) and 18% presented primary hypogonadism (PH). SH was inversely related to HbA1 (partial *r* (rp) = 0.229, *p* < 0.005), while PH was directly related to age (*r* = 0.356, *p* < 0.001). TT levels were reduced independently by metformin (364 ± 160 vs. 431 ± 242 ng/dL, *t* = 2.241, *p* < 0.05) and statins (359 ± 156 vs. 424 ± 230 ng/dl, *t* = 2.224, *p* < 0.05). TT levels were inversely related to microvascular disease (*r*p = −0.169, *p* < 0.05). *Discussion*. TT levels were stable over time and hypogonadism was common. SH, generally clinically, is related to the diabetic state, while PH, generally subclinically, is an age-dependent process unrelated to diabetes. Low TT levels were related to older age, poor metabolic control, metformin and statins use, and microvascular disease.

## 1. Introduction

Diabetes mellitus is a common chronic condition, with a complex and less than optimal treatment, persistent metabolic dysregulation, widespread micro- and macrovascular complications, increased infection susceptibility, bone fractures risk, depressive symptoms, and sleep disturbances, which imposes a significant burden on the patient, family, health services, and society [1–4].

Despite clinical practice recommendations and the chronic care model that empowers patient active self-management in a patient-centered care paradigm, composite targets in multifactorial care are attained in less than 50% of the patients [5]. Multiple targets in a multifactorial disease suggest the importance of exploring general factors of the diabetic state.

Hypogonadism may occur independently of diabetes mellitus [6, 7]. However, hypogonadism may also complicate diabetes mellitus at the very least because of either age or the neuroendocrine adaptation to a chronic condition, but eventually also because of the chronic metabolic derangement, micro- and macrovascular disease, and multiple drug use [8–11]. Hypogonadism further deteriorates metabolic control, cardiovascular risk, and bone and mental health [12–15].

Testosterone presents widespread effects on body composition, metabolism, vascular tone, blood pressure, bone, and behavior, either by itself or after conversion to dihydrotestosterone or estradiol; these effects may occur either by genomic or by nongenomic pathways [16, 17]. Both hypogonadism in males and hyperandrogenism in females result in central obesity, insulin resistance, and hypertension and increase the risk of developing type 2 diabetes mellitus [18–20].

The relation between diabetes mellitus and hypogonadism is therefore complex and difficult to disentangle. However, no formal evaluation of gonadic function is included in current guidelines for diabetes management and the specifics of that evaluation are therefore not discussed [21]. Although a comprehensive medical evaluation is assumed, total testosterone measurement is recommended only in symptomatic patients, with an index of free testosterone for those with borderline values. Case detection of symptomatic individuals rather than screening for low testosterone levels is proposed [21, 22]. However, symptoms of hypogonadism are generally not formally elicited and may be confused with symptoms of the aging process [23].

We systematically explored baseline gonadic function in male persons with diabetes assisted at the outpatient endocrine department of a public central hospital to estimate the frequency of hypogonadism, characterize it, and explore possible relations to diabetic characteristics, pharmacologic drug use, and vascular complications.

## 2. Patients and Methods

A specific SPSS database (27th version IBM SPSS, Inc., New York) containing clinical and analytical data regarding all male persons with diabetes mellitus assisted by one of us at the endocrine outpatient department of a public central and university hospital was defined and used [24, 25]. As previously described, the database includes (1) gender and age; (2) height without shoes and weight without shoes or coats, first and last measurements; the body mass index (BMI) was computed as weight (kg)/height (m)^2^; (3) time since diagnosis; (4) diabetes type defined according to standardized criteria [26]; (5) quality of metabolic control evaluated by the last HbA1c; (6) presence or absence of microvascular disease: retinopathy–last annual ophthalmologic examination; 1—no retinopathy; 2—background retinopathy; 3—laser treated retinopathy; nephropathy—last analytical evaluation; 1—negative microalbuminuria; 2—positive microalbuminuria; 3—positive microalbuminuria and increased serum creatinine; peripheral neuropathy (PN)—last clinical evaluation; 1—no clinical peripheral neuropathy; 2—clinical neuropathy present; autonomic neuropathy (AN)—last clinical evaluation; 1—no clinical autonomic neuropathy; 2—clinical autonomic neuropathy present; a composite index MICRO was computed by adding up previous individual scores; also individual microvascular disease could be dichotomized when indicated as 1—not present and 2—present; (7) presence or absence of macrovascular disease; ischemic heart disease (IHD)—last clinical evaluation; 1—not clinically present; 2—clinically present ischemic heart disease; 3—previous myocardial infarction or revascularization procedure; cerebrovascular disease (CVD)—last clinical evaluation; 1—not clinically present; 2—clinically present; 3—previous stroke or vascular dementia; peripheral vascular disease (PVD)—last clinical evaluation; 1—not clinically present; 2—clinically present; 3—previous lower limb amputation, lower limb ulcer or revascularization procedure; a composite index MACRO was computed by adding up previous scores; again when indicated individual macrovascular could be dichotomized as 1—not present; 2—present; (8) high blood pressure (HBP)—last clinical evaluation or antihypertensive medications in use; 1—not present; 2—present; (9) dyslipidemia—last analytical evaluation or hypolipidemic drug use; 1—not present; 2—present; (10) diabetic medication (pharmacologic class); (11) antihypertensive medication (pharmacologic class); (12) hypolipidemic medication (pharmacologic class); (13) antiplatelet medication (pharmacological class).

At the last appointment, the following was also obtained: total testosterone (TT), FSH, LH, and estradiol (E2). In the last 40 patients, these were repeated at the next two appointments and sequentially defined as TT1, TT2, and TT3.

Patients with known pituitary, testicular, or adrenal disease or current use of medications that affect the pituitary, testicular or adrenal function like corticosteroids, steroids, GnRH agonists/antagonists, inhibitors of 5-alfa-reductase, antagonists of the androgen receptor and anti-psychotics were excluded.

Patients were assisted in the morning period (8–14 h) at the outpatient endocrine department. Venous blood collection took place in the morning period (8–10 h) after the overnight fast in the Clinical Pathology Department of the hospital. All analytical measurements were obtained at that department using commercially available standardized methods, including automated enzymatic methods for glucose and lipids, colorimetric methods for proteins (Roche, cobas 8000, Basel), affinity chromatography for glycated hemoglobin (Hb9210 PremierTM, Trinity Biotech, Bray) and electrochemoluminescence immunoassay (ECLIA) methods for hormone measurements (Roche, cobas 8000, Basel). Intra- and interassay variation coefficients were always below 10%. Reference values for the adult population are established by the Clinical Pathology Department and periodically revised to sustain clinical decisions. For variables used in this report reference values were as follows: HbA1c 4–6%; Cholesterol <190 mg/dL; triglycerides <150 mg/dL; HDLc >40 mg/dL; LDLc <130 mg/dL; TT 240–830 ng/dL; E2 16–60 pg/mL; FSH 2–13 IU/L; LH 2–9 IU/L. The Clinical Pathology Department is certified by the international standard ISO 9001 : 2015 and regularly participates in official quality control programs.

Written informed consent was obtained from all patients regarding the anonymous use of the clinical and analytical data for research purposes, according to the ethical principles for medical research involving human subjects, as defined by the World Medical Association (WMA) (Helsinki declaration available in the WMA official site).

The same SPSS program was used for statistical analysis. Results are presented as the mean ± standard deviation or (%) as appropriate. Normal distribution of continuous variables was verified by the Kolmogorov–Smirnov test and no normal distributed variables were log-transformed prior to analysis or no parametric tests were used. For the sake of simplicity, however, when no differences were found, results regarding the no transformed variables and parametric tests are presented. Differences between groups used the Chi-square test, *t*-Student test, or ANOVA as appropriate. The relation between continuous variables used factorial regression. The limit of statistical significance is 0.05 [27, 28].

## 3. Results

### 3.1. Patients General Characteristics

The database includes 184 male patients with either type 1 or type 2 diabetes, assisted in the 2019-2020 period. Patient general characteristics are presented in Table 1.

### 3.2. Analytical Evaluation of Gonadic Function

**Figure 1:**
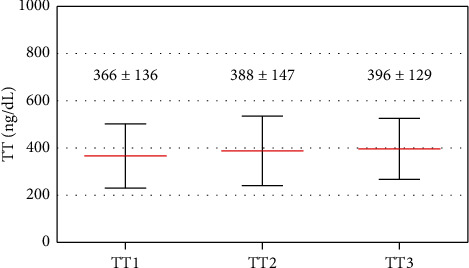
Testosterone levels at the three time moments considered. The mean ± standard deviation is presented.

**Figure 2:**
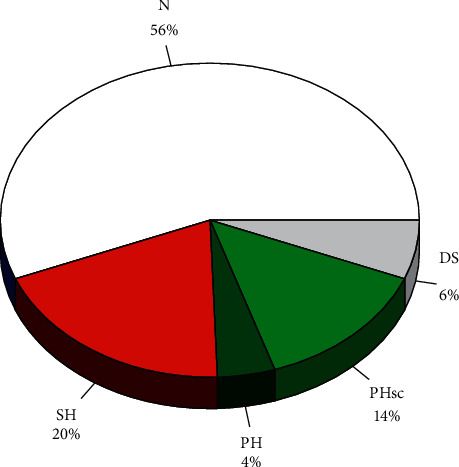
Distribution of diagnostic groups. N: normal gonadic function (normal TT, FSH, and LH); SH: secondary hypogonadism (decreased TT with normal or low LH); PH: primary clinical hypogonadism (decreased TT and increased LH); PHsc: primary subclinical hypogonadism (normal TT with increased LH); DS: defective spermatogenesis (normal TT, normal LH, and increased FSH).

Results regarding the final analytical evaluation of gonadic function and at the three time moments are presented in Figures 1 and 2.

Paired *t*-Student revealed no significant differences regarding the first and the second evaluation, or between the second and the third measurements, except for a small borderline significant increase in TT (combined first and second and second and third evaluations—377 ± 141 ng/dL vs. 393 ± 138 ng/dL, *t* = 1.990, *p* < 0.06). However, between the first and the third measurements there was a significant increase in TT (366 ± 136 ng/dL vs. 396 ± 129 ng/dL, *t* = 2.341, *p* < 0.05) and in E2 (22 ± 10 pg/mL vs. 27 ± 12 pg/mL, *t* = 2.217, *p* < 0.05) and in LH (7 ± 4 IU/L vs. 9 ± 6 IU/L, *t* = 3.533, *p* < 0.01) (Figure 1).

Considering first and second measurements or second and third measurements, TT were highly and directly correlated (combined *r* = 0.788, *p* < 0.001) as were FSH (*r* = 0.819, *p* < 0.001), LH (*r* = 0.654, *p* < 0.001), and E2 (*r* = 0.490, *p* < 0.05). The same occurred regarding first and third measurements: TT (*r* = 0.831, *p* < 0.001), FSH (*r* = 0.839, *p* < 0.001), LH (*r* = 0.925, *p* < 0.001), and E2 (*r* = 0.481, *p* < 0.05).

Regarding the first and second measurements and the second and third measurements, 6/18 (33%) of the patients with initially low testosterone levels would later present normal levels (although low normal in every case) while 4/62 (6%) of the patients with initially normal testosterone levels would later present low levels (although initially low normal in every case). Regarding the first and last measurements, the rates were 50% and 6%, respectively.

At the last measurements, TT was directly and significantly related to E2—*r* = 0.319, *p* < 0.001, but not to FSH or LH. E2 was also not significantly related to either FSH or LH, and both gonadotrophins were directly and significantly related—*r* = 0.748, *p* < 0.001.

Considering last values and reference values, 103 (56%) patients presented normal gonadic function (normal TT, FSH, and LH), 36 (20%) patients presented SH (decreased TT with normal or low LH), 8 (4%) patients presented clinical PH (decreased TT and increased LH), 26 (14%) patients presented subclinical PH (normal TT with increased LH), and 11 (6%) patients presented evidence for defective spermatogenesis (DS) (normal TT, normal LH and increased FSH) (Figure 2).

### 3.3. Characteristics of Patients with Hypogonadism

Patients with SH (decreased TT and normal or low LH) and patients with PH (normal or decreased TT and increased LH) were compared with patients with normal gonadic function (Table 2). As this level of analysis, patients with SH were not older but were more obese, with poorer metabolic control, higher triglyceride levels, and more common PN. Patients with PH were older, with lower LDLc levels and more common nephropathy, PN, HBP, and dyslipidemia.

### 3.4. Primary Independent Variables for Total Testosterone Levels

Multiple linear regression with stepwise analysis revealed that age [partial *r* (*r*p) = −0.260, *p* < 0.001], time since diagnosis (*r*p = +0.273, *p* < 0.001), and last HbA1c (*r*p = −0.229, *p* < 0.005) were independent significant factors for TT together explaining 12% of TT variability. The apparent influence of BMI suggested by comparing SH and normal gonadic function patients occurs because both age (partial *r* (*r*p) = +0.299, *p* < 0.001) and time since diagnosis (*r*p *r* = −0.208, *p* < 0.02) relate to BMI, and the apparent influence of triglycerides occurs because they depend on metabolic control (*r* = +0.155, *p* < 0.05). It should be noted that while age and last HbA1c are inversely related to TT as should be expected, the relation between time since diagnosis and TT is direct, an apparent paradox; however, the older the patient, the longer the time since diagnosis (*r* = +0.333, *p* < 0.001) and the older the patient, the better the metabolic control (partial *r* = −0.238, *p* < 0.001). So part of the paradox is because the longer the time since diagnosis, the older the patient, the better the metabolic control, and therefore the higher the testosterone levels; other reasons will become later evident.

Regarding LH levels, they were directly related to age (*r* = 0.356, *p* < 0.001), but not with time since diagnosis when age was considered, neither to HbA1c, BMI, or Tg. Age accounts for 13% of LH variability.

### 3.5. Drug Effects on Total Testosterone and LH Levels

Patients under statins presented significantly lower TT levels [359 ± 156 (97) vs. 424 ± 230 (87) ng/dl, *t* = 2.224, *p* < 0.05], and the statin effect remained significant even when entering age, years since diagnosis, and last HbA1c (all factors remain significant together explaining 13% of TT variability). Patients under metformin treatment also presented significantly lower TT levels [364 ± 160 (119) vs. 431 ± 242 (65) ng/dL, *t* = 2.241, *p* < 0.05], and again the metformin effect remained significant when age, years since diagnosis, and last HbA1c were introduced in the analysis (all factors remain significant, together explaining 14% of TT levels). The statin and the metformin effects were independent and both remained significant when all other factors were also entered and together explained 15% of TT variability.

Patients under metformin treatment (but not those under statin treatment) presented less time since diagnosis (21 ± 13 years vs. 17 ± 10 years, *t* = 2.249, *p* < 0.05) and this may be another reason why years since diagnosis presented an unexpected protective effect on TT levels. There were no significant differences regarding TT levels in patients using or not angiotensin converting enzyme inhibitors (ACEI), angiotensin receptor blockers (ARB), insulin, or antiplatelet agents.

Patients under statins had no significant higher LH levels (8 ± 7 IU/L vs. 6 ± 5 IU/L, *p* < 0.08) with no significant differences regarding the use of metformin, ACEI/ARB, insulin, or antiplatelet drugs. Statins were no longer a significant factor for LH levels when age was also considered.

### 3.6. Association of TT and LH Levels and Micro- and Macrovascular Disease and Vascular Disease Risk Factors

Results are presented in Table 3. Although patients without microvascular disease—retinopathy, nephropathy, PN, and AN—always presented higher TT values, differences only reached significance regarding PN. The significance of peripheral neuropathy remained when age, years since diagnosis, and last HbA1c were also entered in multiple regression analysis. The composite index MICRO was significantly and inversely related to TT levels when age, time since diagnosis, and last Hba1c were also considered (rp = −0.169, *p* < 0.05).

Patients with evidence of macrovascular disease—ischemic heart disease, cerebrovascular disease, or peripheral vascular disease—always presented lower TT levels, but the difference never reached significance and the composite index MACRO was not significantly related to TT levels.

Regarding LH levels, no differences were found when comparing patients with or without retinopathy or AN, but patients with nephropathy presented significantly higher LH levels (9 ± 7 IU/L vs. 6 ± 3 IU/L, *t* = 3.052, *p* < 0.005) as did those with peripheral neuropathy (9 ± 7 IU/L vs. 6 ± 5 IU/L, *t* = 2.852, *p* < 0.005). However, only nephropathy remained significant when age was also considered. Also although patients with macrovascular diseases—ischemic heart disease, peripheral vascular disease, or cerebrovascular disease—always presented higher LH levels, neither factor remained significant when age was also considered.

Patients with HBP presented lower TT levels but the significance of HBP was no longer evident when age, years since diagnosis, and last HbA1c were entered in multiple regression analysis. By the same token, patients with dyslipidemia presented lower TT levels, but dyslipidemia was no longer a significant factor for TT when statins use was also considered. Patients with HBP or dyslipidemia presented high LH levels, but neither factor remained independently significant for LH levels when age was also considered.

## 4. Discussion

Hypogonadism may complicate diabetes mellitus by several different mechanisms and may worsen metabolic control and macrovascular disease [8–15]. In fact, several studies now suggest the use of testosterone in the treatment of diabetic patients with low testosterone levels to improve body composition and metabolic control, to prevent micro- and macrovascular disease, or even to revert type 2 diabetes mellitus, even if this is still not generally recommended [13, 14, 22, 29–32].

Despite this, characterization of hypogonadism in male diabetic patients remains elusive with no formal guidelines established, with controversies regarding PH or SH, the bidirectional relation between diabetes and hypogonadism, and the relation to vascular disease, as well as indications, objectives, and effectiveness/safety of testosterone therapy [21, 22, 33–36].

This is a clinical study in real setting of a public hospital practice and results can be easily verified and extended by other clinicians. In fact, given the limited number of patients that characterizes by necessity individual practice, the magnitude of any effects must be large enough to reach statistical significance and to be meaningful in the real conditions of clinical practice.

Patients were old (type 2 diabetes mellitus, T2DM) or young (type 1 diabetes mellitus, T1DM) adults, with long-standing disease, overweight, with fair (T2DM) or poor (T1DM) metabolic control, common dyslipidemia (hypercholesterolemia in T1DM and hypertriglyceridemia in T2DM), and controlled blood pressure levels in T2DM. Microvascular disease was also common, mainly the forms that can be easily diagnosed, i.e., retinopathy in both T1DM and T2DM and nephropathy in T2DM because of concurrent high blood pressure, but macrovascular disease was less common, given the poor sensitivity of clinical evaluation, mainly in T2DM. We assume this to be representative of diabetic patients assisted at public central hospitals elsewhere at least in Western Europe [24, 25].

In this presumably representative sample, results of analytical evaluation reveal a distinctive and coherent pattern. It should be noted that standard evaluation of gonadic function should be obtained outside any intercurrent illness, strenuous exercise, or restrictive diet in the fasting state and in the morning and should include total testosterone and an index of free testosterone, like SHBG and albumin or free testosterone itself (less accurate), and should be repeated with at least a three-month interval to confirm low testosterone levels; LH (and FSH) are recommended only later to classify low testosterone levels [21–23, 37].

As suggested by repeated measurements, the hypothalamic-pituitary-testicular axis is rather stable and highly correlated over time (*r* > 0.650, *p* < 0.001) with no significant differences over the 3–6-month period and with only a minor but significant increase over time on TT and FSH and LH that contradicts the TT decrease to be expected with age, over the 6–12-month period [8, 38, 39]. Even if many patients with low testosterone levels will later present normal values (33% and 50%) and less commonly patients with normal testosterone levels will later present low values (6% and 6%), these are, in every case, borderline cases. A high rate of initially low and later normal values has been recognized [22]. So, although repeated measurements are indicated for diagnostic purposes and for therapeutic decisions, clinically these are patients with persistent low or low normal testosterone levels, even if using operative definitions was based on specific limits, patients may be classified with or without hypogonadism on repeated measurements. Interestingly enough, testosterone levels are not related to either FSH or LH, suggesting no general restriction on gonadic function. We do not have a definitive explanation for the slight increase of TT over time, also previously reported [22], although as we will see later time since diagnosis is a protective factor and it would be tempting to assume that it depends on improved metabolic control.

Considering the last analytical evaluation, in this sample of middle and old age persons with diabetes mellitus (62 ± 15 years), almost one-fourth (24%) presented low total testosterone levels and this occurs mainly because of SH (20%), but PH is also common (18%) albeit subclinical (14%) in most cases. Comparing both groups, it seems clear that while SH is a nonage-dependent process clearly related to the diabetic condition, PH is an age-dependent process not related to the diabetic condition. If we further consider DS, a common early marker of testicular dysfunction then PH is even more common than SH although subclinical in most cases. This agrees with other studies [8, 38, 39]; in the European Male Ageing Study, a community-based study that includes 3369 men aged 40–79 years, with mean age 60 ± 19 years, 12% presented SH, 2% clinical PH, and 10% subclinical PH [38]. As shown in that study, the rate of PH but not that of SH increases with age [38]. Although the rates are therefore much higher than those reported for the general population, they are lower than those reported in type 2 diabetes even in younger patients [33].

Assuming low testosterone levels to be the relevant biologic factor, and in this case mostly due to SH, further analysis shows that this is also an age-dependent process, but clearly related to the diabetic state and metabolic control and furthermore with an apparent paradoxical protective effect of time since diagnosis. The apparent protective effect of time since diagnosis may occur because older patients present better metabolic control and longer times since diagnosis, but may also result from the decreased used of metformin in long-standing diabetes (see later). Other authors do not describe this association of testosterone with time since diagnosis or with metabolic control and the difference may occur because of the need to correct for confounding variables and covariation of clinical and analytical markers [33–39].

The relation to obesity, suggested by comparing patients with SH and patients with normal gonadic function, may depend on the influence of both age and time since diagnosis on BMI. However, other authors report a significant effect for BMI independent of age [33–39]; again these differences may result from covariation of several factors or from a more restricted BMI range in this report, but it should be noted that diabetic patients with SH do not present increased estradiol concentrations, a possible link between obesity and SH [33–36]. Also although insulin resistance has been proposed as yet another mechanism for the association of type 2 diabetes and SH, we found that metformin that corrects insulin resistance is associated with decreased testosterone levels [33–36]. More probably, inflammatory mediators can contribute to the central suppression of the hypothalamic-pituitary-gonadic axis and these may be increased in obese diabetic subjects but also in old, long-standing, and complicated diabetic subjects without obesity [33].

Both statins and also metformin are independently associated with low testosterone levels, but not with increased LH levels. Statins reduce testosterone levels and DHEAS, despite reducing inflammation and inflammatory markers; interestingly enough, statins also increase the risk of developing type 2 diabetes [40–42]. Although some reports describe a 3-4% decrease of TT levels, we found a much larger effect of around 15% similar to that previously described in women [40–42]. Interestingly enough, this much larger effect may also result because of the lowering effect of metformin. We are aware of only one previous report regarding a lowering effect of metformin on testosterone levels in males [43]. However, metformin reduces testosterone levels in women with insulin resistance and the polycystic ovary syndrome [44] and pioglitazone also reduced testosterone levels in male persons with type 2 diabetes mellitus [45]. Furthermore, no such effect was found for insulin, other hypoglycemic drugs, or in relation to ACEI/ARB agents or antiplatelet drugs. As noted, the paradoxical protective effect of time since diagnosis factor apparently results at least in part from less metformin use with longer disease.

On the contrary, increased LH levels due to primary hypogonadism mostly subclinical is only an age-dependent process not related to the diabetic condition.

Low testosterone levels, but not increased LH levels, are also associated with microvascular disease—decreased levels in patients with retinopathy, nephropathy, peripheral, and autonomic neuropathy—although the difference only reaches significance for peripheral neuropathy and the composite MICRO index. Low testosterone levels, but again not increased LH levels, are also associated with macrovascular disease—decreased levels in patients with ischemic heart disease, cerebrovascular disease, and peripheral vascular disease—but differences never reach statistical significance, not even when considering the composite MACRO index; this may depend at least in part on the low rate of these complications and the crude nature of clinical evaluation. Of course statistical associations do not indicate causality, which may be bidirectional. Nonetheless, the association of low testosterone levels and microvascular but not with macrovascular disease suggests microvascular disease is another factor for SH, and this may be another reason for the striking association between peripheral neuropathy and low testosterone levels. To our knowledge, only one report describes the association of lower testosterone and microvascular disease [46], contrary to the well-known association with macrovascular disease [47]; however, it is well known that diabetic microvascular disease generally occurs after puberty and some reports show that microvascular disease in type 1 diabetics is more common in the female sex [33–36, 48, 49].

## 5. Conclusions

In short, we found that, in diabetic patients, hypogonadism is common, but different conditions must be distinguished, although this may be difficult because of covariation of clinical and analytical factors. One-fifth of these patients present SH with low testosterone levels, which is clearly related to the diabetic condition, namely, metabolic control, metformin, and statin use, and the presence of microvascular disease. Almost another fourth present PH, generally subclinical that is an age-dependent process not related to the diabetic state. Low testosterone levels in diabetic patients are related to the diabetic state and may be improved by significant weight loss and better metabolic control, selective drug use, and preventing microvascular disease.

## Figures and Tables

**Table 1:** General characteristics of the patients.

	T1DM (*n* = 38)	T2DM (146)	*p*
Age (years)	41 ± 12	67 ± 11	*t* = 12.438, *p* < 0.001
Time since diagnosis (years)	21 ± 13	18 ± 11	Ns
Insulin use (%)	100	55	χ^2^ = 25.904, *p* < 0.001
BMI (kg/m^2^)	24.4 ± 3.8	29.1 ± 4.2	*t* = 5.433, *p* < 0.001
HbA1c (%)	8.7 ± 1.7	7.8 ± 1.6	*t* = 2.957, *p* < 0.005
Cholesterol (mg/dL)	184 ± 51	160 ± 34	*t* = 3.385, *p* < 0.001
Triglycerides (mg/dL)	112 ± 73	143 ± 82	*t* = 2.126, *p* < 0.05
HDLc (mg/dL)	55 ± 14	45 ± 14	*t* = 3.465, *p* < 0.001
LDLc (mg/dL)	107 ± 42	86 ± 28	*t* = 3.659, *p* < 0.001
SBP (mmH g)	125 ± 20	134 ± 18	*t* = 2.514, *p* < 0.05
DBP (mmHg)	70 ± 15	70 ± 11	Ns
Retinopathy (yes) (%)	44	45	Ns
Cataracts (yes) (%)	5	27	χ^2^ = 7.653, *p* < 0.05
Nephropathy (yes) (%)	29	56	χ^2^ = 8–056, *p* < 0.005
PN (yes) (%)	14	37	χ^2^ = 7.445, *p* < 0.01
AN (yes) (%)	8	6	Ns
HBP (yes) (%)	30	90	χ^2^ = 69.462, *p* < 0.001
Dyslipidemia (yes) (%)	24	62	χ^2^ = 16.556, *p* < 0.001
IHD (yes) (%)	5	20	χ^2^ = 4.328, *p* < 0.05
CVD (yes) (%)	3	13	χ^2^ = 3.188, *p* < 0.08
PVD (yes) (%)	0	19	χ^2^ = 8.096, *p* < 0.005
TT (ng/dL)	495 ± 252	361 ± 168	*t* = 3.901, *p* < 0.001
E2 (pg/mL)	23 ± 14	26 ± 13	Ns
FSH (IU/L)	6 ± 4	10 ± 9	*t* = 2.159, *p* < 0.05
LH (IU/L)	6 ± 5	8 ± 6	Ns

T1DM: type 1 diabetes mellitus; T2DM: type 2 diabetes mellitus; BMI: body mass index [weight (kg)/height (cm)^2^]; HbA1c reference values (RV) 4–6%; cholesterol RV < 190 mg/dL; triglycerides RV < 150 mg/dL; HDLc RV > 40 mg/dL; LDLc RV < 130 mg/dL; SBP: systolic blood pressure RV < 140 mmH; DBP: diastolic blood pressure RV < 90 mmHg; TT RV 240–830 ng/dL; E2 RV 16–60 pg/mL; FSH RVs 2–13 IU/L; LH RV 2–9 IU/L.

**Table 2:** Comparisons between patients with normal gonadic function (*n*) and those with secondary hypogonadism (sh) or primary hypogonadism (ph).

	SH (*n* = 36)	*p*	*N* (*n* = 103)	*p*	PH (*n* = 34)
Age (years)	60 ± 16	ns	60 ± 15	*t* = 3.809, *p* < 0.01	72 ± 10
Years since diagnosis	16 ± 10	ns	19 ± 12	ns	21 ± 9
Type 1/2 (%)	13/87	ns	24/76	ns	10/90
Insulin use (yes) (%)	53	ns	63	ns	76
BMI (kg/m^2^)	29.4 ± 5.2	*t* = 2.007, *p* < 0.05	27.6 ± 3.8	ns	28.9 ± 5.0
HbA1c (%)	8.4 ± 2.0	*t* = 1.974, *p* < 0.05	7.7 ± 1.6	ns	7.7 ± 1.4
Cholesterol (mg/dL)	163 ± 33	ns	165 ± 35	ns	153 ± 53
Triglycerides (mg/dL)	165 ± 89	*t* = 3.197, *p* < 0.01	121 ± 60	ns	147 ± 121
HDLc (mg/dL)	44 ± 14	ns	48 ± 13	ns	46 ± 19
LDLc (mg/dL)	85 ± 29	ns	93 ± 30	*t* = 2.233, *p* < 0.05	77 ± 39
SBP (mmhg)	131 ± 14	ns	132 ± 21	ns	135 ± 19
DBP (mmHg)	69 ± 10	ns	70 ± 13	ns	70 ± 12
Retinopathy (yes) (%)	48	ns	48	ns	46
Cataracts (yes) (%)	13	ns	20	ns	39
Nephropathy (yes) (%)	58	ns	42	χ^2^ = 6.640, *p* < 0.01	70
PN (yes) (%)	39	χ^2^ = 4.299, *p* < 0.05	22	χ^2^ = 14.129, *p* < 0.01	59
AN (yes) (%)	5	ns	5	Ns	10
HBP (yes) (%)	87	ns	75	χ^2^ = 2.728, *p* < 0.1	90
Dyslipid (yes) (%)	58	Ns	47	χ^2^ = 4.156, *p* < 0.05	69
IHD (yes) (%)	21	Ns	14	Ns	14
CVD (yes) (%)	11	Ns	10	Ns	17
PVD (yes) (%)	18	Ns	12	Ns	24
TT (ng/dL)	199 ± 67	*t* = 8.340, *p* < 0.01	447 ± 177	*t* = 2.003, *p* < 0.05	370 ± 193
E2 (pg/mL)	23 ± 13	Ns	25 ± 13	Ns	26 ± 13
FSH (UI/L)	6 ± 4	Ns	5 ± 3	*t* = 9.068, *p* < 0.01	20 ± 13
LH (UI/L)	5 ± 2	Ns	5 ± 2	*t* = 11.972, *p* < 0.01	17 ± 8

**Table 3:** Association between TT levels and micro- and macrovascular disease.

Retinopathy (N vs. Y)	(101) 403 ± 221	(83) 387 ± 168	ns
Nephropathy (N vs. Y)	(93) 414 ± 214	(91) 363 ± 158	*t* = 1.678, *p* < 0.1
PN (N vs. Y)	(125) 411 ± 211	(59) 337 ± 148	*t* = 2.431, *p* < 0.02
AN (N vs. Y)	(173) 389 ± 196	(11) 321 ± 134	ns
MICRO (none vs. any)	(48) 433 ± 237	(136) 383 ± 167	ns

IHD (N vs. Y)	(153) 392 ± 198	(31) 367 ± 179	ns
CVD (N vs. Y)	(164) 392 ± 201	(20) 353 ± 140	ns
PVD (N vs. Y)	(156) 397 ± 197	(28) 333 ± 182	*t* = 1.620, *p* < 0.15
MACRO (none vs. any)	(118) 404 ± 213	(66) 359 ± 157	*t* = 1.458, *p* < 0.15

HBP (N vs. Y)	(40) 455 ± 282	(144) 370 ± 159	*t* = 2.480, *p* < 0.02
Dyslipidemia (N vs. Y)	(84) 425 ± 201	(100) 358 ± 185	*t* = 2.363, *p* < 0.02

## Data Availability

The research database is available on request from the first author (jmartinmartins@sapo.pt).

## Additional Points

Secondary but not primary hypogonadism is related to the diabetic state. Metformin and statins reduce total testosterone levels. Total testosterone is inversely related to metabolic control and microvascular disease.
